# Peripheral Regulatory Cells Immunophenotyping in Kidney Transplant Recipients with Different Clinical Profiles: A Cross-Sectional Study

**DOI:** 10.1155/2012/256960

**Published:** 2012-11-19

**Authors:** Janette Furuzawa-Carballeda, Guadalupe Lima, Perla Simancas, Dolores Ramos-Bello, Margaret Simancas, Ian C. Bostock, Mario Vilatobá, Bernardo Gabilondo, Julio Granados, Luis Morales-Buenrostro, Josefina Alberú, Luis Llorente

**Affiliations:** ^1^Department of Immunology and Rheumatology, National Institute of Medical Sciences and Nutrition, 14000 Mexico City, DF, Mexico; ^2^Department of Transplantation, National Institute of Medical Sciences and Nutrition, 14000 Mexico City, DF, Mexico; ^3^Department of Nephrology, National Institute of Medical Sciences and Nutrition, 14000 Mexico City, DF, Mexico

## Abstract

Regulatory Foxp3-expressing T cells (Tregs), IL-10-producing B cells (Bregs), and IDO-expressing dendritic cells (DCregs) downregulate inflammatory processes and induces peripheral tolerance. These subpopulations also might participate in maintaining allograft immunological quiescence in kidney transplant recipients (KTRs) with an excellent long-term graft function under immunosuppression (ELTGF). The aim of the study was to characterize and to enumerate peripheral Tregs, Bregs, and DCregs in KTR with an ELTGF for more than 5 years after transplant. Fourteen KTR with an ELTGF, 9 KTR with chronic graft dysfunction (CGD), and 12 healthy donors (HDs) were included in the study. CD19^+^-expressing peripheral B lymphocytes were purified by positive selection. IL-10-producing B cells, CD4^+^/CD25^hi^, and CD8^+^/CD28^−^ Tregs, as well as CCR6^+^/CD123^+^/IDO^+^ DCs, were quantitated by flow cytometry. IL-10-producing Bregs (immature/transitional, but not CD19^+^/CD38^hi^/CD24^hi^/CD27^+^B10 cells), CCR6^+^/CD123^+^/IDO^+^ DCs, and Tregs from ELTGF patients had similar or higher percentages versus HD (*P* < 0.05). By contrast, number of Tregs, DCregs, and Bregs except for CD27^+^B10 cells from CGD patients had lower levels versus HD and ELTGF patients (*P* < 0.05). The findings of this exploratory study might suggest that in ELTGF patients, peripheral tolerance mechanisms could be directly involved in the maintenance of a quiescent immunologic state and graft function stability.

## 1. Introduction

Progress in elucidating cellular, molecular, and biochemical processes that regulate immune response provides increasingly plausible explanations for the normal status of tolerance to self-antigens that guards most humans from Ehrlich's imagined horror autotoxicus [1]. Emerging data on regulatory antigen-presenting cells (APCs) provide fertile ground for resolving some perplexing immunological paradoxes. One specific mechanism that appears to play a key role is the catabolism of tryptophan, by the enzyme indoleamine 2,3-dioxygenase (IDO) [2, 3].

IDO is upmodulated during antigen presentation by the engagement of CTLA-4/B7.1/B7.2 (CD80/CD86) molecules on lymphocytes and human dendritic cells (DCs), in response to infection and tissue inflammation (TNF-*α*, PGE_2_, IFN-*α*/*β*/*γ* secretion) [2–4].

IDO generates kynurenines, 3-hydroxyanthranilic, and quinolic acids, molecules with the ability to induce T-cell apoptosis and to exert cytotoxic action on T, B, and NK cells, but not on DCs themselves [5, 6]. IDO has a selective sensitivity of Th1 over Th2 cells to tryptophan metabolites, suggesting a potential role for Th2 differentiation [7]. Furthermore, deprivation of tryptophan by IDO halts the proliferation of T cells at mid-G_1_ phase, which in concert with the proapoptotic activity of kynurenine and leads to diminishing T cell-mediated immune responses and the subsequent development of immune tolerance [6, 8, 9].

In addition, IDO-competent DCs have shown to induce CD4^+^/CD25^+^ regulatory T cells (Tregs) *in vivo* and Treg-expressed glucocorticoid-induced tumor necrosis factor receptor (GITR) which in turn can use IDO^+^ DCs to expand their own population in a positive feedback loop [10, 11]. Thus, IDO-producing cells might play a role in preventing the initiation of autoimmune disorders and transplant rejection [9, 12–14].

Alternatively, Treg cell-mediated suppression serves as a vital mechanism of negative regulation of immune-mediated inflammation and features prominently in autoimmune and autoinflammatory disorders, allergy, acute and chronic infections, cancer, and metabolic inflammation. Tregs have also shown to have a pivotal role in transplant tolerance leading to graft acceptance and prevention of rejection in xenotransplantation [15]. Tregs have primary effect on T cells and/or DCs by three main regulatory modes of action including cell-to-cell contact [16]; competition for growth factors (local effect), expression of soluble factors (IL-10, IL-35, IL-9, and TGF-*β*) with direct suppressive effects on T cells [17, 18], fibrinogen-like protein-2 and granzyme B with apoptotic effects on T cells, and prevention of maturation of DCs [19]; production of adenosine by CD39/73 cleavage of ATP, which causes cell cycle arrest in T cells and prevention of maturation and decreased APC capability in DCs by binding to the A2A receptor [20].

A third tolerogenic mechanism, that has recently shown to contribute to the maintenance of the fine equilibrium required for peripheral tolerance, comprises one functional IL-10-producing B cell subset. The immunoregulatory role of B cells in autoimmune disease was characterized in B cell-deficient mice immunized with a myelin basic protein peptide in complete Freund's adjuvant, where mice develop exacerbate encephalomyelitis compared to controls [21]. This Breg subset differentiate in a chronic inflammatory environment, express high levels of CD1d, produce IL-10, and suppress the progression of intestinal inflammation by directly downregulating inflammatory cascades associated with IL-1*β* and STAT3 activation [22, 23]. Lately, it has been described a CD19^+^CD24^hi^CD38^hi^ B cell subset that suppress the differentiation of Th1 cells in an IL-10-dependent, but TGF-*β*-independent manner, which requires CD80/CD86 interactions with target CD4^+^ T cells. In addition, these Bregs favor the differentiation and maintenance of Foxp3-expressing Tregs and may control organ specific inflammation [23, 24]. Therefore, B regulatory mechanisms include regulation through effector molecules such as IL-10 and TGF-*β* produced after stimulation via CD40, TLR, or BCR; production of protective antibodies that binds to CD32 on DCs and suppression of APC function and/or neutralization of self-antigens; suppression of antigen presentation through the production of IL-10 or CXCL13 or negative regulation of TCR crosslinking of CD4^+^ T cells; activation of CD1d by iNKT cells; regulation of mucosal-associated lymphoid tissue activation of cytotoxic CD8 cells [19–24].

Several studies in kidney transplant recipients (KTRs) with operational tolerance have reported a direct relationship between soluble and cellular tolerance mechanisms and the presence of Foxp3-expressing Tregs and IDO-producing DCregs. It is noteworthy, that recent evidence indicates that Bregs might enhance tolerance [25–28]. 

The aim of this study was to characterize and to enumerate peripheral IL-10-producing B cell subpopulations, Foxp3-expressing CD4^+^/CD25^+^ and CD8^+^/CD28^−^ T cells, as well as IDO-producing CCR6^+^/CD123^+^ DCs from KTR with ELTGF for more than 5 years after transplant.

## 2. Material and Methods

### 2.1. Patients

This study was an exploratory, observational, and cross-sectional clinical trial that included 23 KTR patients. They were allocated in 2 groups: 14 KTR patients with ELTGF and 9 KTR patients with chronic graft dysfunction (CGD). Twelve healthy donors (HD) age-matched were included as controls. The protocol was approved by the Committee of Medical Ethics (Reference number 2022) and performed in accordance with the revised Declaration of Helsinki content. All patients gave informed consent to participate. 

### 2.2. Definitions and Key Inclusion Criteria

ELTGF patients were defined as having ≥5 years after transplant, serum creatinine (sCr) ≤1·2 mg/dL, estimated glomerular filtration rate (eGFR) by modified diet in renal disease (MDRD) formula ≥60 mL/min, absence of albuminuria, 24-hour proteinuria ≤150 mg, and immunosuppressive regimen with azathioprine ≤100 mg/day and/or prednisone ≤5 mg/day.

CGD patients were defined as having ≥5 years after transplant, sCr ≥1·5 mg/dL, eGFR by MDRD ≤50 mL/min, on triple drug immunosuppressive regimen based on calcineurin inhibitor (CNI) (cyclosporine/tacrolimus) or motor (Sirolimus), an antiproliferative drug (azathioprine/mycophenolate mofetil), and prednisone. 

### 2.3. Key Exclusion Criteria

Patients with a previous graft biopsy with evidence of primary renal disease recurrence or *de novo* glomerulopathy; patients with acute deterioration of graft function due to biopsy proven acute cellular or antibody mediated rejection (Banff′03) during the previous 12 months; patients with acute systemic or localized inflammation of the urinary tract by infection or obstruction, history of any malignancy, presence of chronic infection by HCV/HBV, and multiorgan transplant recipients were excluded from the study. 

### 2.4. Peripheral Blood Mononuclear Cells (PBMCs) Isolation

A 100 mL sample of venous blood was obtained from each subject. PBMCs were isolated by gradient centrifugation on Lymphoprep (Axis-Shield PoC AS, Oslo, Norway).

### 2.5. B Cell Purification and Cytometric Analysis

CD19-mAb-coated microbeads (Miltenyi Biotec, Bergisch Gladbach; Germany) were used to purify blood B cells by positive selection following the manufacturer's instructions.

#### 2.5.1. Flow Cytometry

CD19^+^ cells were surface stained with several combinations of antihuman fluorochrome-conjugated antibodies for four color analysis. CD19^+^ cells were stained with 5 *μ*L of anti-CD38-PECy5-labeled, anti-CD38-PE-conjugated, anti-CD24-FITC-labeled, anti-IgA-PE-conjugated, anti-IgD-PE-labeled, anti-IgG-PECy5-conjugated, anti-IgM-APC-labeled, anti-CD5-APC-conjugated, anti-CD10-APC-labeled, anti-CD20-APC-conjugated, anti-CD27-APC-labeled, anti-CXRC4-APC-conjugated, and anti-CXCR7-Cy5-labeled monoclonal antibodies (BD Biosciences, San Jose, CA, USA). Cells were stained for intracellular IL-10 with PE-conjugated-anti-IL-10 or FITC-labeled-anti-IL-10 (BD Biosciences). Finally, CD19^+^ subsets were analyzed by flow cytometry with a FACScalibur (BD Biosciences). An electronic gate was made for CD38^hi^, IgA^+^, IgD^+^, IgG^+^, or IgM^+^, and IL-10^+^ or for CD19^+^/CD38^hi^, CD5^+^, CD10^+^, CD20^+^, CD27^+^, CXCR4^+^, or CXCR7^+^, and IL-10^+^ cells, and a total of 30,000–50,000 events were recorded for each sample and analyzed with the CellQuest Pro software (BD Biosciences). Results are expressed as the relative percentage of IL-10-expressing B cells in each gate. As isotype controls, IgG_1_-FITC/IgG_1_-PE/CD45-PeCy5 mouse IgG_1_, k (Figures 2(c)–2(e)) (BD Tritest, BD Biosciences) and PE-conjugated-anti rat-IL-10 IgG (Figure 2(b)) (BD Biosciences) were used to set the threshold and gates in the cytometer. We ran an unstained (autofluorescence control) and permeabilized PBMCs sample (Figure 2(a)). Autofluorescence control (unstained cells) was compared to single stained cell positive controls to confirm that the stained cells were on scale for each parameter. Besides, BD Calibrite 3 beads were used to adjust instrument settings, set fluorescence compensation, and check instrument sensitivity (Figures 2(c)–2(e)) (BD Calibrite, BD Biosciences). 

To determine IDO cell expression, non-B cells were labeled with an anti-human CCR6-PE and CD123-PECy5 monoclonal antibodies (BD Biosciences). Cells were stained with a sheep anti-human-IDO (Chemicon, Temecula, CA, USA) and then with FITC-conjugated-rabbit antisheep antibody. Cell subset was analyzed by flow cytometry. As control of FITC-labeled-rabbit antisheep specificity staining, cells were incubated with surface antibodies and FITC-conjugated-rabbit antisheep in the absence of sheep anti-human IDO antibody (Figures 3(k) and 3(l)). An electronic gate was made for each and every one of the surface markers employed. Results are expressed as the relative percentage of IDO-expressing cells in each gate. 

For Tregs, non-CD19^+^ B cells were conjugated with an anti-human CD4-FITC and CD25-PECy5 or CD8*α*-FITC and CD28-PECy5 (BD Biosciences). Intracellular staining was performed with an anti-human Foxp3-PE-labeled (BD Biosciences) monoclonal antibody. An electronic gate was made for CD4^+^/CD25^hi^ cells or CD8^+^/CD28^−^. Results are expressed as the relative percentage of Foxp3-expressing cells in each gate.

**Figure 1:**
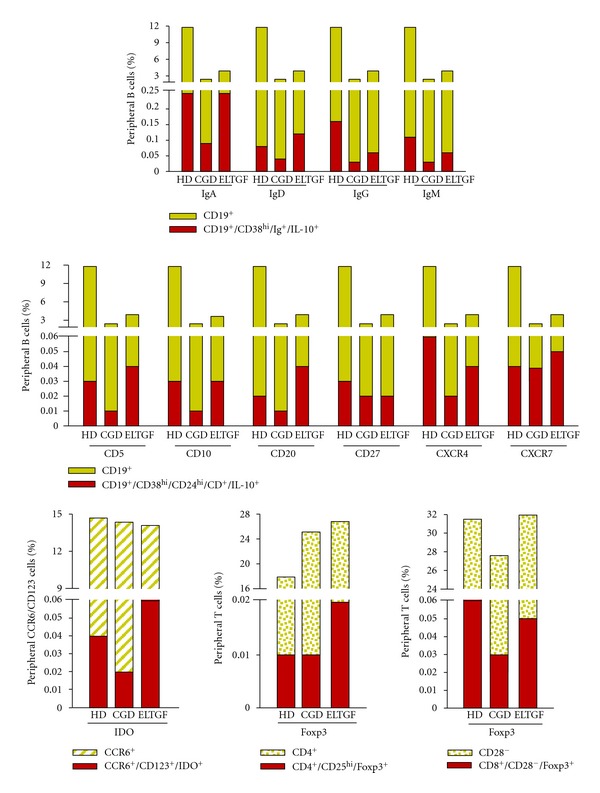
Percentage of IL-10-producing B cells, IDO^+^-, and Foxp3^+^-circulating cells were calculated from percentage of CD19^+^, CCR6^+^, CD4^+^, or CD28^−^ cells, respectively, obtained following positive selection. HD: healthy donor, CGD: chronic graft dysfunction, and ELTGF: excellent long-term graft function under immunosuppression.

Percentage of IL-10-producing B cells, IDO^+^- and Foxp3^+^-circulating cells were calculated from percentage of CD19^+^, CCR6^+^, CD4^+^, or CD28^−^ cells, respectively, obtained following the positive selection (Figure 1).

### 2.6. Statistics

Statistical analysis was performed using the SigmaStat11*·*2 program (Aspire Software International, Leesburg, VA, USA) by the Kruskal-Wallis one way analysis of variance on ranks and by Holm-Sidak for all pairwise multiple comparison procedures. Data were expressed as the median, range, and mean ± s.d./s.e.m. The *P* values smaller than or equal to 0*·*05 were considered as significant. 

## 3. Results

### 3.1. Demographic and Clinical Data

Demographic, clinical, and laboratory characteristics of the patients are summarized in Table 1. Initial immunosuppressive regimen included cyclosporine/azathioprine/prednisone in 3 (21%) ELTGF patients; the remaining 11 (79%) ELTGF patients received azathioprine/prednisone only since the initial post-KT course, with current mean doses of 82.5 ± 23.7 mg/day and 4.8 ± 0.8 mg/day, respectively. It is worth mentioning that 2 ELTGF patients withdrew immunosuppression *motu proprio* at 1 and 3 years posttransplant and have remained stable without it for 25 and 16 years, respectively (operational tolerant). In addition, immunosuppression was withdrawn in another ELTGF patient, 13 years after transplantation during hospitalization for fever, headache, and brain MRI lesions suggestive of a posttransplant lymphoproliferative disorder. Patient has remained off immunosuppression since then and is currently in her 14th year posttransplant. 

In 6 (67%) patients from CGD group, triple drug immunosuppression scheme consisted of CNI (tacrolimus, mean blood level 6.0 ± 2.9 ng/mL, cyclosporine, mean blood level 71.2 ± 27.2 ng/mL), mycophenolate mofetil (mean daily dose 1.0 g), and prednisone (mean daily dose 5.0 mg). The remaining 3 (33%) patients received sirolimus (mean blood level 8.3 ± 3.7 ng/mL), mycophenolate mofetil (mean daily dose 1.0 g), and prednisone (mean daily dose 5.0 mg).

### 3.2. Biopsies

Three graft biopsies from 3 different ELTGF patients were performed at 6, 16, and 21 years posttransplant. Unspecific findings such as mild CNI toxicity, mild interstitial fibrosis, and interstitial fibrosis and tubular atrophy of less than 15% were observed, respectively. 

Twenty four biopsies from 9 CGD patients were performed during posttransplant followup. A history of acute cellular rejection Banff IB and acute humoral rejection grade I was found in biopsies from 2 different patients. These latter patients received treatment with methylprednisolone boluses (*n* = 1), plasmapheresis, IVIg, methylprednisolone boluses, and bortezomib (*n* = 1). 

Overall, evidence of CNI and interstitial fibrosis and tubular atrophy was found in 57%, and data suggestive of chronic rejection was observed in 78% of patients from the CGD group. 

It is important to highlight that currently, eGFR is doubled in ELTGF *versus* CGD group (Table 1). Also, the immunosuppressive regimen is more intensive in patients with CGD.

### 3.3. Surface Expression of Immunoglobulin on IL-10-Producing B Peripheral Cell Subtypes

**Figure 2:**
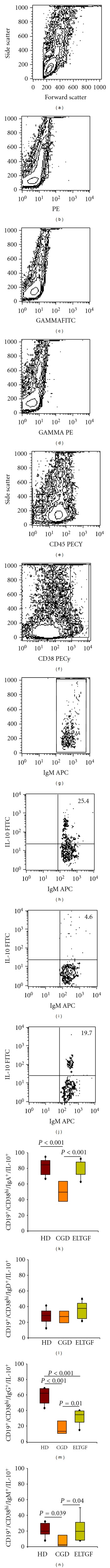
Percentage of immunoglobulin expression on IL-10-producing B peripheral cell subtype in KTR. (a) Autofluorescence control. (b) PE-conjugated-anti-rat-IL-10 IgG isotype control. (c–e) IgG_1_-FITC/IgG_1_-PE/CD45-PeCy5 mouse IgG_1_,k isotype controls. (f) Representative contour plot of CD19^+^ B cells from a patient. An electronic gate was made for CD38^hi^ cells. (g) From the gate *f* CD19^+^/CD38^hi^/IgM^+^ cells were determined. From the latter CD19^+^/CD38^hi^/IgM^+^/IL-10^+^, cells were defined in (h) a healthy donor (HD), (i) a chronic graft dysfunction (CGD) patient, and (j) an excellent long-term graft function under immunosuppression (ELTGF) patient. A total of 30,000–50,000 events were recorded for each sample before any gate setting and analyzed with the CellQuest Pro software (BD Biosciences). Bar graphs show percentage of (k) IgA^+^, (l) IgD^+^, (m) IgG^+^, and (n) CD19^+^/CD38^hi^/ IL-10^+^/IgM^+^ cells. Results are expressed as median, 10th, 25th, 75th, and 90th percentiles.

To enumerate the frequency of surface expression of immunoglobulin on IL-10-producing B peripheral cells in KTR with CGD and ELTGF, B19^+^ cells were stained and analyzed (Figures 2(f)–2(j)). The results showed that the percentages of IgA- and IL-10-producing B cells were equivalent in HD and ELTGF patients, and they were ≅38% and ≅39% higher for HD and ELTGF versus CGD patients (*P* < 0.001; Figure 2(k), Table 2). In contrast, cell number of IgG- and IL-10-expressing B cells were ≅46% higher in HD *versus* ELTGF patients (*P* < 0.001), and the latter two groups had ≅70% and ≅45% higher positive cells, respectively, versus CGD patients (*P* ≤ 0.01; Figure 2(m), Table 2). Percentages of IgM- and IL-10-producing B cells were equivalent in HD and ELTGF patients, and they were ≅68% and ≅70% higher, for HD and ELTGF *versus* CGD patients (*P* = 0.04; Figure 2(n), Table 2).

No differences were found in IgD- and IL-10-producing B circulating cell percentage among CGD and ELTGF patients and controls (Figure 2(l), Table 2). 

### 3.4. Immature/Transitional IL-10-Producing B Peripheral Cell Subtypes

**Figure 3:**
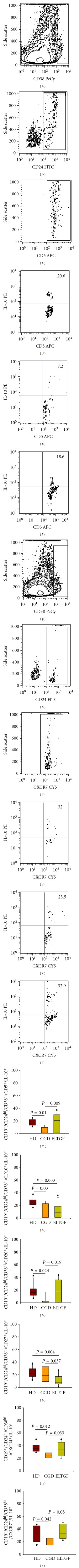
Percentage of immature/transitional IL-10-producing B peripheral cell subtype in KTR. (a) Representative contour plot of CD19^+^ B cells from a patient. An electronic gate was made for CD38^hi^ cells. (b) From the gate *a* CD19^+^ *⁄*CD38^hi^ *⁄*CD24^hi^ cells were determined. (c) From the gate *b* CD19^+^/CD38^hi^/CD24^hi^/CD5^+^ were defined and an electronic gate was made for positive cells. From the latter CD19^+^/CD38^hi^/CD24^hi^/CD5^+^/IL-10^+^ cells were determined in (d) a healthy donor (HD), (e) a chronic graft dysfunction (CGD) patient, and (f) an excellent long-term graft function under immunosuppression (ELTGF) patient. (g) Percentage of homing receptor-expressing Breg subtype in KTR. An electronic gate was made for CD38^hi^ cells. (h) From the gate *g* CD19^+^/CD38^hi^/CD24^hi^ cells were determined. (i) From the gate *h* CD19^+^/CD38^hi^/CD24^hi^/CXCR7^+^ were defined. From the latter CD19^+^/CD38^hi^/CD24^hi^/CXCR7^+^/IL-10^+^ cells were determined in (j) a HD, (k) a CGD, and (l) an ELTGF. Bar graphs show percentage of (m) CD5^+^, (n) CD10^+^, (o) CD20^+^, (p) CD27^+^, (q) CXCR4^+^, and (r) CD19^+^/CD38^hi^/CD24^hi^/CXCR7^+^/IL-10^+^-producing B peripheral cells. A total of 30,000–50,000 events were recorded for each sample before any gate setting and analyzed with the CellQuest Pro software (BD Biosciences). Results are expressed as median, 10th, 25th, 75th, and 90th percentiles.

Frequencies of CD19^+^/CD38^hi^/CD24^hi^/CD5^+^/IL-10^+^ and CD19^+^/CD38^hi^/CD24^hi^/CD20^+^/IL-10^+^ were similar in controls and ELTGF, and they were between ≅75% and ≅83% higher versus CGD patients (*P* ≤ 0.02; Figures 3(a)–3(f), 3(m), and 3(o), Table 2). It is noteworthy that, percentages of CD19^+^/CD38^hi^/CD24^hi^/CD10^+^/IL-10^+^ were lower in ELTGF (≅50%) and in CGD patients (≅66%) *versus* controls (*P* ≤ 0.03; Figure 3(n), Table 2).

### 3.5. CD27^+^B10 Cell Subset

A different IL-10 B subset, a nonmemory B cell that could be the human counterpart of mouse marginal zone B cells, CD19^+^/CD38^hi^/CD24^hi^/CD27^+^B10 subset was immunophenotyped. B10 subset was conspicuously decreased in ELTGF (≅54%) and CGD patients (≅47%) *versus* controls (*P* ≤ 0.04; Figure 3(p), Table 2).

### 3.6. Homing Receptor-Expressing Immature/Transitional Breg Cell Subtypes

CD19^+^/CD38^hi^/CD24^hi^/CXCR4^+^/IL-10^+^ cell percentages were akin in HD and ELTGF patients; however, the latter two groups had ≅32% and ≅27% higher levels *versus* CGD patients (*P* ≤ 0.03; Figure 3(q), Table 2). CD19^+^/CD38^hi^/CD24^hi^/CXCR7^+^/IL-10^+^ B cell percentages were comparable in HD and ELTGF patients; nonetheless the latter two groups had ≅42% higher levels versus CGD patients (*P* ≤ 0.04; Figures 3(g)–3(l), and 3(r), Table 2).

### 3.7. IDO-Expressing Peripheral Blood Cells

**Figure 4:**
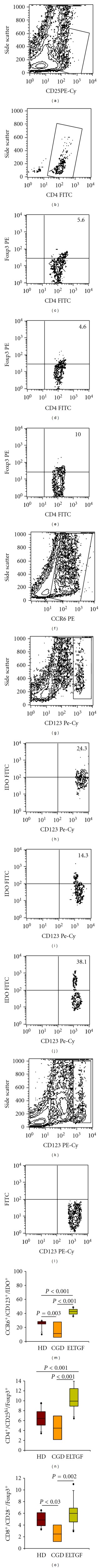
Percentage of IDO-and Foxp3-expressing peripheral blood cells in KTR. (a) Representative contour plot from a patient. An electronic gate was made for CD25^hi^ cells. (b) From the gate *a*, CD4^+^/CD25^hi^ were determined, and an electronic gate was made for double positive cells. From the latter, CD4^+^/CD25^hi^/Foxp3^+^ cells were defined in (c) a healthy donor (HD), (d) a chronic graft dysfunction (CGD), and (k) an excellent long-term graft function under immunosuppression (ELTGF) patient. (f) An electronic gate was made for CCR6^+^ cells. (g) From the gate *f*, CCR6^+^/CD123^+^ cells were determined, and an electronic gate was made for double positive cells. From the latter, CCR6^+^/CD123^+^/IDO^+^ cells were defined in (h) a HD, (i) a CGD patient, and (j) an ELTGF patient. (k, l) Control of FITC-conjugated-rabbit antisheep specificity staining. Percentage of (m) CCR6^+^/CD123^hi^/IDO^+^, (n) CD4^+^/CD25^hi^/Foxp3^+^, and (o) CD8^+^/CD28^−^/Foxp3^+^ peripheral blood cells. A total of 50,000 events were recorded for each sample before any gate setting and analyzed with the CellQuestPro software (BD Biosciences). Results are expressed as median, 10th, 25th, 75th, and 90th percentiles.

IDO^+^ DCs had ≅65% higher percentage in ELTGF patients *versus* controls and CGD (*P* < 0.001), whilst CGD had ≅40% lower levels of IDO-circulating cells *versus* controls (*P* = 0.003; Figures 4(f)–4(j) and 4(m), Table 2).

### 3.8. Foxp3-Expressing T Peripheral Blood Cells

CD4^+^/CD25^hi^ Treg frequency in ELTGF patients was ≅38% higher versus controls and ≅57% higher versus CGD patients (*P* < 0.001; Figures 4(a)–4(e) and 4(n), Table 2); whereas, CD8^+^/CD28^−^ Treg percentage was ≅44% lower in CGD patients *versus* HD (*P* < 0.030) and ≅53% lower *versus* ELTGF patients (*P* = 0.002; Figure 4(o) Table 2).

## 4. Discussion

The present study depicts the immunophenotype of some peripheral tolerogenic cell subsets in KTR with excellent long-term allograft function compared to patients with CGD. 

Besides several regulatory T cells, our results show that human peripheral blood has at least 2 more tolerogenic subsets, namely, DCregs and IL-10-secreting Bregs. Blair and colleagues have defined these latter cells as a regulatory B cell pool with many subtypes that display a CD19^+^/CD24^hi^/CD38^hi^ phenotype [23]. Recently, a CD19^+^/CD24^hi^/CD38^hi^/CD5^hi^ B cell subtype has been described. It suppresses the proliferation of Th1 through CD40 engagement and STAT-3 phosphorylation. Meanwhile, the differentiation of Th1 cells is suppressed in an IL-10-dependent, but TGF-*β*1-independent manner, which requires CD80/CD86 interactions with target, CD4^+^ T-cells. In addition to halting Th1 but not Th17 responses, the suppressive effects are mediated by an indirect mechanism, through the induction of Foxp3^+^ expression in CD4^+^/CD25^+^ T cells [23, 24] in a more efficiently way than any other population of APCs [25, 26]. The resulting Tregs displayed a greater suppressive capacity than regulatory T-cells generated by immature DCs from the same donor [27]. It suggests that B cell-dependent suppressive effects are associated with the generation of Foxp3-expressing CD4^+^/CD25^+^ Tregs. Our results in KTR with ELTGF under 2-drug immunosuppression are in keeping with those previously reported in KTR patients, who did not require continuous immunosuppressive therapy and have different subsets of suppressive cells, including higher proportions of CD19^+^/CD24^hi^/CD38^hi^/CD5^+^ IL-10-secreting B cells compared to those patients with CGD [25–28].

Another B cell subpopulation in our patients was CD19^+^/CD38^hi^/CD24^hi^/CD10^+^/IL-10^+^ cells. CD10 is a cell membrane metallopeptidase expressed by early B, pro-B, and pre-B lymphocytes and diffuse large B cells. CD10 expression is a well-accepted marker for most cells within the transitional B-cell pool, its absence on CD19^+^/CD24^+^/CD38^+^/IL-10^+^ cells suggests that these cells are not recent emigrants from the bone marrow [23]. Our findings show a statistically significant decrease in frequency of this regulatory B cell subtype in ELTGF and CGD patients compared to HD, attributable to a more mature differentiation stage of these cells (probably T2) [29].

The higher CD19^+^/CD38^hi^/CD24^hi^/CD20^+^/IL-10^+^ percentage of Breg subtype in ELTGF patients compared to an almost imperceptible cell number in CGD patient's correlates with the findings of Newell et al., related to identification of a B cell signature in renal transplant tolerance [25]. CD20 is a 33 kd phosphoprotein similar to an ion channel that allows calcium influx for cell activation. It is expressed on pre-B and mature B cells after CD19/CD10 expression and before CD21/CD22 and surface immunoglobulin expression. It is retained on mature B cells until plasma cell development (plasmablasts) [25]. 

In addition to human CD19^+^/CD24^hi^/CD38^hi^-circulating B cell subpopulation, it has been suggested that B10 might be a different Breg subset. It is present in the splenic marginal zone rather than memory cells generated in germinal centers. Whereas CD40/CpG-stimulated B10 cells induce proliferation and produce higher levels of IL-10 compared to CD27^−^, only B10 cells inhibit mitogen-induced TNF-*α* production by monocytes, via IL-10 synthesis [30–35]. B10 cell subset is markedly increased in ELTGF patients compared to CGD, suggesting neither a noninflammatory nor an infectious process.

Among all chemokine receptors, CXCR4 possesses a unique response profile and distinguishes itself through prolonged signaling capacity. Upon stimulation, CXCR4 induces the prolonged activation of intracellular signal transduction pathways, such as MAPK cascade. This may elicit antiapoptotic responses and thus, contribute to cell survival. In B cell lymphopoiesis, CXCR4/CXCL12 is critical for bone marrow retention and maturation of the cells [36]. Meanwhile, CXCR7 or RDC1 expression correlates with the capacity to differentiate into plasma cells upon polyclonal activation. Moderate RDC1 expression is observed on pro-B and pre-B cells and becomes gradually upregulated during development into the relative immature/transitional B cell state. RDC1 is essential for survival and differentiation to the switch to memory cells [37]. The expression of CXCR4 and CXCR7 in CD19^+^/CD38^hi^/CD24^hi^/IL-10^+^ cells suggests that Bregs in patients with ELTGF could migrate to the site of inflammation and may perhaps interact *in situ* with proinflammatory cells.

The role of IDO-expressing cells in normal and disease conditions has not yet been fully characterized. Munn et al. have demonstrated that DC maturation does not in and of itself abrogate IDO-mediated suppression, allowing some DCs to manifest a phenotype that is both immature and actively suppressive [8]. Generally, less than 3% of the IDO-circulating cell subpopulation is sufficient to promote immune suppression, directly or by means of bystander suppression [14]. CD123^+^/CCR6^+^/IDO^+^ plasmacytoid DCs (pDCs) constitute only 0.2–0.8% of peripheral blood cells and represent a unique, rather plastic, versatile and important immune cell population capable of producing over 95% of IFN-I synthesized by PBMCs in response to viruses, as well as nucleic acid-containing complexes from the host [8, 38]. IFN-*α* secretion is indispensable for high-level expression of IDO after B7.1/B7.2 ligation to CTLA-4 [39]. IDO-expressing pDCs contributes to Tregs generation from CD4^+^/CD25^−^ T-cells through cell-cell interaction (ICOSL-ICOS), with compelling suppressor cell function [40]. Thus, IDO^+^ DCregs are relevant not only due to its *per se* ability to induce immune suppression through tryptophan catabolism, but also in the context of providing a regulatory bridge that connects two independent T-cell populations, namely, the effector T-cells, and the Foxp3^+^ Tregs from naïve CD25^−^ T cells after exposure to combined tryptophan depletion and kynurenine excess [41]. In this vein, KTR with ELTGF show higher frequencies of CD123^+^/CCR6^+^/IDO^+^-circulating pDCs compared to HD and CGD.

Lastly, Tregs are diverse populations of lymphocytes that regulate immune response, delete autoreactive T-cells, induce tolerance, and dampen inflammation. Foxp3-expressing CD8^+^/CD28^−^ Tregs share developmental and phenotypic features (CD122^+^/GITR^+^/CTLA4^+^/CCR7^+^/CD62L^+^/CD25^+^/CD127^−^/IL-23R^−^) with naturally occurring CD4^+^ Tregs. Secretion of IL-10 and TGF-*β*1 is higher in CD8^+^/CD25^+^ Tregs than in CD8^+^/CD25^−^ T-cells. In addition, Foxp3-expressing CD8^+^ Tregs reduce T cell proliferation in response to a specific antigen and secretion of both IFN-*γ* and IL-17 by CD4 T cells. On the other hand, CD8^+^ Treg cells down-regulate the expression of costimulatory molecules on DCs (CD40, CD80, CD86, MHC I, and HLA-DR) leading to a less efficient antigen presentation. Moreover, it has been shown that CD8 Tregs activate IDO in DCs [42–44]. KTR with an ELTGF shows higher frequency of CD8^+^/CD28^−^/Foxp3^+^ Tregs compared to CGD. However, KTR with an ELTGF have highest level of CD4^+^/CD25^hi^/Foxp3^+^ Tregs compared to HD and CGD patients. 

Interestingly, ELTGF patients display significantly increased numbers of IL-10-secreting Bregs, DCregs and CD4^+^, and CD8^+^ Tregs compared to patients who require more intense immunosuppressive therapy to sustain graft function. Thus, it is not preposterous to speculate that notwithstanding its reduced absolute numbers, the regulatory peripheral cell subpopulations of KTR with an ELTGF may play a critical role in the regulation of the allograft acceptance. 

The cellular regulatory findings detected in ELTGF patients of this study occurred under immunosuppression for the majority of them. This fact might suggest that the mechanisms underlying the development of a regulatory pattern are not abrogated, at least with the combination of azathioprine and prednisone. Certainly, no significant differences were detected between the patients off immunosuppression and the remaining patients of the ELTGF group who receives variable doses of azathioprine. On the other hand, 67% of the patients included in the CGD group have been chronically under a CNI as part of their immunosuppressive scheme. Hence, CNIs are not expected to induce a “tolerogenic” state.

Several issues regarding peripheral tolerance in KTR with CGD remain unexplained that deserves consideration: Why are Tregs, Bregs, and DCregs numerically different when compared to patients with ELTGF? Could the numerical deficiency of IL-10-producing B cells, Tregs, and DCregs in CGD patients be due to treatment or a *per se* patient condition? Have these cells lost their capacity to suppress inflammation? Or maybe, are the inflammatory mechanisms more aggressive in CGD patients than in ELTGF patients? Is the elevated number of Tregs, Bregs, and DCregs in ELTGF patients a signature that is a consequence of immunologic quiescence or the cause of it? Certainly many more questions could be posed regarding the biology of inflammation/tolerance mechanisms in humans. However, what seems to be conclusive from our results is that KTR with an ELTGF has a similar frequency of IL-10-secreting Bregs and CD8^+^/CD28^−^/Foxp3^+^ Tregs compared to HD, whereas IDO-expressing DCs and CD4^+^/CD25^hi^/Foxp3^+^ Tregs have higher percentages compared to HD. 

**Figure 5:**
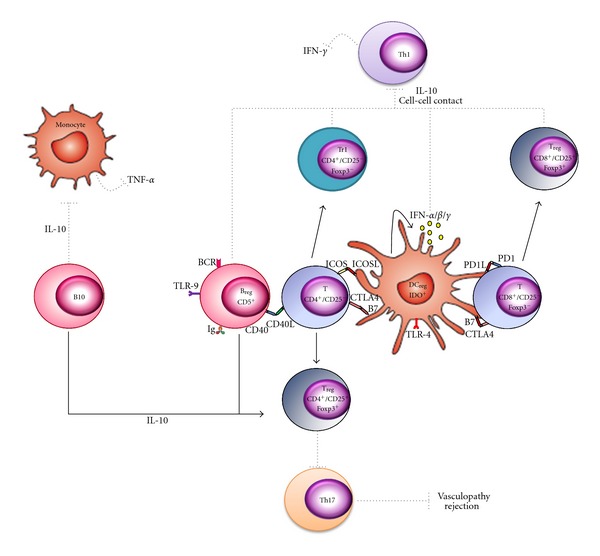
Hypothetical model by which Bregs, Tregs, and DCregs generate a positive feedback in a three-way loop. IL-10—producing Breg cells favor the differentiation of CD4^+^/CD25^−^ T cells into CD4^+^/CD25^+^ Foxp3-expressing T cells or CD4^+^/CD25^+^/Foxp3^−^ T cells after stimulation through CD40/CD40L or B7.1,B7.2/CTLA-4, respectively, in presence of IL-10. IL-10 induces phosphorylation of STAT3 (pSTAT3) in Foxp3—expressing CD4^+^ and CD8^+^ Tregs, Tr1 and DCs. Tregs with pSTAT3 are capable of suppressing Th17 responses or activation of STAT1 and induce/upregulate IDO activity in DCs through mechanisms requiring CTLA-4 expression, whereas Tr1 suppresses Th1 response and IFN-*γ* production by cell-cell contact and/or IL-10 production. On the other hand, pSTAT3 in DCs induces downregulation of antigen presenting cell function (CD40, CD80, CD86, MHC I, and HLA-DR expression). Moreover, activated DCs by IFN-*α*/*β*/*γ* express IDO after B7.1/B7.2 ligation to CTLA-4. IDO-expressing DC cells, as Bregs, contribute to regulatory T cell generation (CD4^+^/CD25^+^/Foxp3^+^) from CD4^+^/CD25^−^ T cells through ICOSL-ICOS interaction. Meanwhile, IDO-expressing DC cells contribute to differentiate CD8^+^/CD25^+^/Foxp3^+^ Tregs from CD8^+^/CD25^−^ T cells through PD1-PDL1/2 interaction. CD19^+^/CD24^hi^/CD38^hi^/CD27^+^ B10 cells suppress TNF-*α* production by monocytes, via IL-10 expression.

Renal transplant patients with CGD (except for B10 cells) have lower frequency of regulatory cells and in consequence, of regulatory mechanisms of peripheral tolerance. These Bregs, Tregs, and DCregs subsets might actively participate as a compensatory mechanism to develop peripheral tolerance in transplant patients suppressing inflammatory processes, through a positive feedback loop of a three-way interaction between Bregs-Tregs-DCregs (Figure 5). 

Much remains to be learned about tolerance mechanisms. Next evaluation should consider exploring ontogeny and population diversity, differentiation pathways, master gene regulator(s), specific surface markers, plasticity, and functionality of cells involved.

Our preliminary results thus, deserve to be studied in depth in order to evaluate the clinical relevance of these findings.

## Figures and Tables

**Table 1:** Demographic and clinical data of kidney transplant recipients.

	ELTGF (*n* = 14)	CGD (*n* = 9)
Demographics		
Age at KT (years)		
Mean ± SD	34.7 ± 11.6	34.0 ± 10.0
Range	(17–60)	(22–51)
Current age (years)		
Mean ± SD	56.0 ± 9.6	43.0 ± 11.0
Range	43–70	31–60
Gender (female/male)	8/6	1/8
Type of donor (LRD/ DD)	12/2*	7/2*
Donor age at KTR (years)		
Mean ± SD	32.9 ± 9.33	39.9 ± 13.0
Range	(19–50)	(20–61)
Donor gender (female/male)	7/7	8/1

Clinical		
Cause of ESRD	A = 8, B = 1, C = 1, D = 2, E = 1, F = 1	A = 4, C = 1, D = 1, G = 2
Time elapsed since transplant (years)		
Mean ± SD	21.3 ± 5.6	9.2 ± 2.4
Range	7–30	4–12
Histocompatibility (Hap-match)	2-Hap = 8; 1-Hap = 4; 0-Hap = 2	1-Hap = 5; 0-Hap = 4
Current immunosuppression regimen (patients)	AZA/PDN = 11	CNI/MMF/PDN = 6 SRL/MMF/PDN = 3
Without immunosuppression (patients)	3	0

Laboratory		
sCr 1 year post-KT, (mg/dL)		
Mean ± SD	1.06 ± 0.39	1.75 ± 0.43
Range	0.55–1.7	1.26–2.81
sCr current, (mg/dL)		
Mean ± SD	0.90 ± 0.21	1.84 ± 0.21
Range	0.55–1.20	1.57–2.26
1 year post-KT eGFR (mL/min)		
Mean ± SD	72.00 ± 25.7	47.00 ± 5.90
Range	50–134	40–57
Current eGFR (mL/min)		
Mean ± SD	79.00 ± 20.3	38.00 ± 7.23
Range	60–126	23–48
ΔGFR = current – 1 year post-KT, (mL/min)		
Mean ± SD	8.0 ± 32.4	−9.00 ± 7.00

HD: healthy donor, CGD: chronic graft dysfunction, ELTGF: excellent long-term graft function under immunosuppression, LRD: living related donor, DD: deceased donor, cause of ESRD: A: unknown, B: congenital, C: chronic UTI, D: Glomerulonephritis, E: Poststreptococcal glomerulonephritis, F: Nonsteroidal anti-inflammatory drugs, G: Diabetes Mellitus, sCr: serum creatinine, ESRD: end stage renal disease, eGFR: estimated glomerular filtration rate, AZA: azathioprine, PDN: prednisone, CNI: calcineurin inhibitor, MMF: mycophenolate mofetil, SRL: sirolimus.

*DD was assigned as “0-Haplotypes-match” because donor MHC typing was not available.

**Table 2:** Percentage of IL-10-producing B cells, IDO- and Tregs-circulating cells in KTR patients.

	HD (*n* = 12)	CGD (*n* = 9)	ELTGF (*n* = 14)	ELTGF under treatment (*n* = 11)	ELTGF w/o treatment (*n* = 3)
CD19-expressing B cells (%)					
CD19^+^					
Mean ± s.e.m.	11.8 ± 1.1	2.4 ± 1.4	3.9 ± 0.5		
Median	12.5	1.5	3.0		
Range	6.5–15.5	0.1–7.8	2.0–7.5		
IL-10-producing B cells (%)					
CD19^+^/CD38^hi^/**IgA** ^+^					
Mean ± s.e.m.	81.9 ± 2.8	49.8 ± 5.9	80.3 ± 3.0	80.4 ± 2.9	80.0 ± 8.9
Median	84.5	49.5	83.4	82.9	85.0
Range	66.7–94.9	30.8–66.7	62.8–92.3	67.7–88.2	62.8–92.3
CD19^+^/CD38^hi^/**IgD** ^+^					
Mean ± s.e.m.	27.7 ± 2.8	26.9 ± 3.6	36.0 ± 3.1	36.8 ± 3.9	34.0 ± 6.2
Median	28.6	27.4	37.7	40.0	35.0
Range	12.2–41.4	15.4–37.2	21.6–50.0	21.6–50.0	22.8–44.23
CD19^+^/CD38^hi^/**IgG** ^+^					
Mean ± s.e.m.	58.4 ± 3.0	17.2 ± 4.1	31.4 ± 2.9	31.4 ± 3.5	31.2 ± 6.2
Median	62.2	13.0	34.6	33.9	35.3
Range	43.0–68.8	7.4–34.3	14.8–40.0	14.8–40.0	19.1–39.3
CD19^+^/CD38^hi^/**IgM** ^+^					
Mean ± s.e.m.	21.2 ± 2.5	6.8 ± 3.4	22.7 ± 4.9	21.2 ± 5.4	26.1 ± 12.41
Median	19.9	2.6	19.6	19.4	20.0
Range	7.9–32.2	1.0–21.4	8.0–50.0	8.0–50.0	8.3–50.0
CD19^+^/CD38^hi^/CD24^hi^/**CD5** ^+^					
Mean ± s.e.m.	17.6 ± 1.4	4.3 ± 1.8	17.6 ± 3.5	17.0 ± 3.9	20.1 ± 9.8
Median	16.7	1.0	20.6	18.6	25.8
Range	11.3–25.0	1.0–13.6	1.0–37.5	1.0–37.5	1.0–33.3
CD19^+^/CD38^hi^/CD24^hi^/**CD10** ^+^					
Mean ± s.e.m.	25.3 ± 1.9	8.7 ± 3.9	11.3 ± 3.0	8.9 ± 2.6	20.6 ± 10.3
Median	23.9	1.0	9.1	9.1	25.0
Range	16.7–37.5	1.0–26.8	1.0–35.7	1.0–29.7	1.0–35.7
CD19^+^/CD38^hi^/CD24^hi^/**CD20** ^+^					
Mean ± s.e.m.	17.9 ± 3.0	3.1 ± 1.8	18.3 ± 4.3	17.8 ± 4.8	20.3 ± 11.3
Median	16.05	1.0	17.1	14.2	20.0
Range	7.1–43.0	1.0–17.1	1.0–44.0	1.0–44.0	1.0–40.0
CD19^+^/CD38^hi^/CD24^hi^/**CD27** ^+^					
Mean ± s.e.m.	23.6 ± 2.3	20.7 ± 4.1	10.9 ± 2.2	9.0 ± 2.3	18.1 ± 3.7
Median	21.9	18.5	8.0	6.3	16.7
Range	11.8–37.9	8.0–36.4	1.0–27.1	1.0–27.1	12.5–25.0
CD19^+^/CD38^hi^/CD24^hi^/**CXCR4** ^+^					
Mean ± s.e.m.	36.9 ± 1.9	25.0 ± 1.8	34.5 ± 3.0	35.9 ± 3.7	30.1 ± 5.1
Median	35.2	25.0	33.3	35.4	33.3
Range	29.5–50.0	17.2–33.3	20.0–50.0	20.0–50.0	20.0–36.8
CD19^+^/CD38^hi^/CD24^hi^/**CXCR7** ^+^					
Mean ± s.e.m.	33.6 ± 3.7	19.6 ± 3.9	34.0 ± 3.3	31.1 ± 3.0	41.7 ± 8.3
Median	40.0	22.5	33.3	30.2	50.0
Range	14.6–47.1	1.0–27.3	22.2–50.0	22.2–47.8	25.0–50.0
IDO-expressing cells (%)					
**CD123** ^hi^/**CCR6** ^+^/IDO^+^					
Mean ± s.e.m.	25.1 ± 1.6	14.9 ± 4.4	42.8 ± 1.2	42.3 ± 1.5	44.3 ± 1.2
Median	26.7	11.3	42.8	42.1	44.4
Range	10.2–29.3	3.7–29.2	36.0–48.7	36.0–48.7	42.2–46.2
Foxp3-expressing T cells (%)					
**CD4** ^+^/CD25^hi^/Foxp3^+^					
Mean ± s.e.m.	6.5 ± 0.5	4.5 ± 0.9	10.4 ± 0.7	10.5 ± 0.8	10.1 ± 1.5
Median	6.4	4.5	9.9	9.8	10.1
Range	3.5–9.5	1.9–7.0	6.5–14.1	6.5–14.1	7.6–12.6
**CD8** ^+^/CD28^-^/Foxp3^+^					
Mean ± s.e.m.	5.0 ± 0.3	2.8 ± 0.7	6.0 ± 0.6	6.5 ± 0.6	4.7 ± 1.4
Median	4.9	2.5	6.0	6.5	3.7
Range	3.3–6.5	1.0–6.0	2.9–11.0	4.4–11.0	2.9–7.4

## Abbreviations

APCs: Antigen-presenting cells
APC: Allophycocyanin
Bregs: IL-10-producing B cells
CGD: Chronic graft dysfunction
CNI: Calcineurin inhibitors
CpG: Cytosine-phosphate-guanine
ELTGF: Excellent long-term graft function under immunosuppression
DC: Dendritic cell
DCregs: IDO-expressing dendritic cells
eGFR: Estimated glomerular filtration rate
ESRD: End stage renal disease
FITC: Fluorescein isothiocyanate
g: Gram (s)
GITR: Glucocorticoid-induced tumor necrosis factor receptor
HD: Healthy donors
HCV: Hepatitis C virus
HBV: Hepatitis B virus
ICOS: Inducible costimulatory molecule
ICOSL: Inducible costimulatory molecule ligand
IDO: Indoleamine 2,3-dioxygenase
iNKT: Inducible natural killer T cells
IVIg: Intravenous immunoglobulin
KTR: Kidney transplant recipients
MAPK: Mitogen-activated protein kinase
MDRD: Modified diet in renal disease
mg: Milligram (s)
MHC: Major histocompatibility complex
mL: Milliliter (s)
MRI: Magnetic resonance imaging;
ng: Nanogram (s)
PBMC: Peripheral blood mononuclear cells
PE: Phycoerythrin
PE Cy5: Phycoerythrin and Cyanine 5
pDCs: Plasmacytoid dendritic cells
sCr: Serum creatinine
SD: Standard deviation
SEM: Standard error of the mean
Th: T helper
TLR: Toll-like receptor
Tregs: Regulatory Foxp3-expressing T cells.
